# Prevalence of tick-borne encephalitis virus (TBEV) RNA in *Dermacentor reticulatus* ticks from natural and urban environment, Poland

**DOI:** 10.1007/s10493-014-9836-5

**Published:** 2014-07-22

**Authors:** Beata Biernat, Grzegorz Karbowiak, Joanna Werszko, Joanna Stańczak

**Affiliations:** 1 Department of Tropical Parasitology, Institute of Maritime and Tropical Medicine, Medical University of Gdansk, Powstania Styczniowego 9B Street, 81-519 Gdynia, Poland; 2 W. Stefański Institute of Parasitology, Polish Academy of Sciences, Twarda 51/55 Street, 00-818 Warsaw, Poland

**Keywords:** *Dermacentor reticulatus*, TBEV, Tick-borne encephalitis virus, nRT-PCR, Poland

## Abstract

Tick-borne encephalitis virus (TBEV) (Flaviviridae, *Flavivirus*) is an arthropod-borne virus, an etiologic agent of tick-borne encephalitis (TBE), a human infection involving the central nervous system. The disease is endemic in a large region in Eurasia, where it is transmitted mainly by *Ixodes ricinus* and *Ixodes persulcatus* ticks. It is known that also *Dermacentor* *reticulatus* is involved in circulation of TBEV. However, the current knowledge of *D. reticulatus* importance in TBE epidemiology is still insufficient. A total of 471 adult *D.* *reticulatus* ticks were collected by flagging vegetation in the Białowieża Primeval Forest, Biebrza National Park, Masurian Landscape Park (North-Eastern Poland) and in the city of Warsaw in the years 2007–2010. All collected ticks were examined individually for the presence of RNA of TBEV using nested RT-PCR assay. Positive results were noted in all investigated localities with the infection rate ranging from 0.99 to 12.5 % with a total mean of 2.12 %. The difference in the percentage of infective males and females was not statistically significant.

## Introduction

Tick borne encephalitis (TBE) is a viral zoonosis caused by TBE virus (TBEV) belonging to the tick-borne flavivirus group, family Flaviviridae, genus *Flavivirus*. This is the most important tick-transmitted arbovirus of human pathogenicity in Eurasia. TBE is an endemic disease in a zone extending from central and eastern Europe to Siberia and Japan which corresponds to the distribution of the ixodid ticks *Ixodes* *ricinus* and *Ixodes persulcatus* which act both as the vectors and the reservoir of TBEV. In recent years, the range of TBEV distribution has been growing, especially in north-western Europe. This phenomenon is associated with global warming leading to increased activity of ticks (Grey 2008; Qviller et al. 2014). The ornate dog tick *Dermacentor reticulatus* (Amblyommidae) occurs in the temperate climate zone, across Eurasia from England and France to Yenisei River basin in Siberia (Russia). Its area of occurrence is divided into two regions—the Western European region, ranging from France to the eastern part of Germany, and the Eastern European region, ranging from eastern Poland, through Belarus and European part of Russia to Siberia (Karbowiak and Kiewra 2010; Siuda 1995). The expansion of this species is observed in north-western Europe. A study performed in Germany by Dautel et al. (2006) showed that the distribution and abundance of *D. reticulatus* on deer and vegetation has increased. This species has also been found in the Netherlands and Belgium (Nijhof et al. 2007; Cohez et al. 2012) and colonization of new sites was observed also in Slovakia (Bullová et al. 2009) and Czech Republic (Široký et al. 2011). In Poland, *D. reticulatus* occurs mostly in north-eastern part of the country, although western migration of this species has been observed since the nineties of the XX century (Karbowiak and Kiewra 2010) and recently it has been found also in Upper and Lower Silesia (SW Poland) (Cuber et al. 2013; Kiewra and Czułowska 2013). Even though the role of *I. ricinus* in TBEV transmission in Europe as well as TBEV infection level of these ticks is becoming better understood (Hubálek and Rudolf 2012; Stefanoff et al. 2013), there is significantly less data concerning *D. reticulatus*. Studies on *D. reticulatus* and other ticks of this genus experimentally infected with TBEV show proliferation of this virus in the ticks (Alekseev et al. 1996; Řeháček et al. 1987). Kožuch and Nosek (1971) confirmed *D. reticulatus* ticks as possible TBEV vector but this species exhibits lower transmission rates (Grešíková and Kaluzová 1997). Other authors just generally report this species as an occasional and competent vector for TBEV (Georgiev et al. 1971; Randolph et al. 1996). The research conducted on 20 *Dermacentor* spp. ticks in Austria did not give results positive for the presence of TBEV (Dobler et al. 2008). Although a later study showed the infection rate with TBEV found in *D. reticulatus* ticks in eastern Poland amounted to 10.8 % and was considerably larger compared to rate found in *I. ricinus* ticks (1.6 %) (Wójcik-Fatla et al. 2011). A tick can be infected in any active stage of development and due to transstadial and transovarial transmission, every stage may transmit infections to mammals. *Dermacentor reticulatus* is characterized by a broad range of hosts which changes with longitude accordingly to the local fauna. In Poland, adult *D. reticulatus* attack mostly wild Cervidae including elks (*Alces alces*), whereas in the Białowieża Primeval Forest also European bison (*Bison bonasus*), on whose bodies they can even overwinter (Karbowiak et al. 2003). Elks were until recently considered to be the main hosts of *D. reticulatus* in Poland due to overlapping coverage of both species (Kadulski 1989). More recent reports indicate equally important role of Cervidae (Bogdaszewska 2005). Despite many years of studies, this species has been found on wild boar (*Sus scrofa*) only occasionally (Kadulski 1989; Fryderyk 1998). Other hosts are also domestic cattle, goats, sheep and dogs (Karbowiak et al. 2003; Zahler et al. 2000; Zygner and Wiśniewski 2006). There are no reports of this tick species parasitizing horses in Poland. Infrequently *D. reticulatus* has been found on human skin (Bursali et al. 2013; Estrada-Peña and Jongejan 1999) including cases noted in north-eastern Poland (Bartosik et al. 2011; Biernat 1995—unpublished observation). Humans acquire TBEV infection by the bite of an infected tick or by consumption of infected, raw (unpasteurized) milk of goat, less commonly sheep or cow or dairy products (Balogh et al. 2010; Grešíková 1972). Over the past decades, TBE has become a growing public health concern in Europe and Asia and is the most important viral tick-borne disease in Europe (Süss 2011). In the recent years, since the sudden and not entirely explained increase in the incidence of TBE in 1993, Poland has recorded yearly from 101 (in 1999) to 260 (in 2008) new cases of this disease. Over 90 % of them were reported in voivodeships: Podlaskie, Warmińsko-Mazurskie, Mazowieckie, Dolnośląskie and Opolskie (Stefanoff et al. 2006, 2008) but little is known about the occurrence of TBEV in native *D. reticulatus* populations. The objective of this study was a determination of the infection level with TBEV of *D. reticulatus* ticks collected from vegetation in north-eastern Poland, the endemic area of TBE and urban environment of the capital city of Warsaw (SC Poland).

## Materials and methods

### Tick collecting

**Fig. 1:**
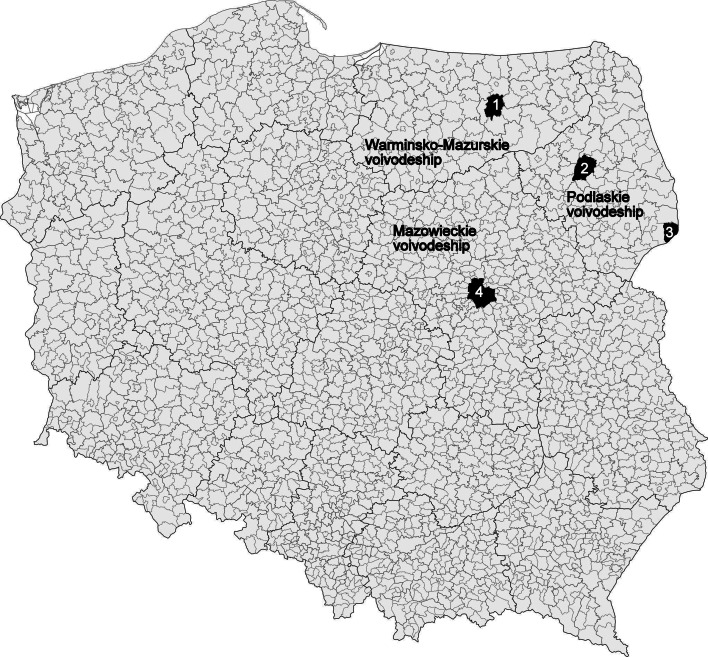
Tick collecting areas. Districts: 1—Mrągowo, 2—Trzcianne, 3—Białowieża, 4—Warsaw

Questing adult *D. reticulatus* ticks (females and males) were collected by flagging the lower vegetation (grassy areas) in the years 2007–2010. Ticks were collected in four localities in north-eastern Poland: (1) Kosewo Górne (Masurian Landscape Park, Mrągowo district, Warmińsko-Mazurskie voivodeship), (2) Gugny, Trzcianne (Biebrza National Park, Trzcianne district, Podlaskie voivodeships), (3) Białowieża Primeval Forest (Białowieża National Park, Białowieża district, Podlaskie voivodeships) and (4) Warsaw (the area in the vicinity of Wał Miedzeszyński street, running on a flood embankment along the right bank of Vistula River, Mazowieckie voivodeships) (Fig. 1). Ticks were sorted according to the collecting site and sex, identified to the species level using the key in a monographic work of Siuda (1993), placed individually in plastic vials and frozen in −80 °C for further investigation.

### RNA extraction

Ticks were homogenized individually in Fenozol (A&A Biotechnology, Gdynia, Poland) with glass pearls using Ultra Turrax Tube Disperser (IKA, Germany). Total RNA was subsequently extracted by the phenol–chloroform method according to the A&A Biotechnology protocol and the obtained templates were kept frozen in −80 °C for further investigation. RNA extraction was conducted in a sterile, nuclease-free environment, in a laminar flow safety cabinet class II (Safeflow 1.2 BioAir, EuroClone Division).

### Nested RT-PCR (reverse transcription-polymerase chain reaction)

#### Reverse transcription reaction

The reverse transcription reactions were performed as described previously (Huang et al. 2001). To prepare cDNA, 5 µl of RNA, 5 µl of 0.5 mM dNTPs mixture (MBI Fermentas), 2.5 µl (0.8 µg) of random hexamer primers (Invitrogen, USA) and 8 µl of RNase-free water (A&A Biotechnology) were heated at 99 °C for 5 min, cooled to 4 °C for 5 min and then was added the reverse transcription (RT) reaction mixture containing: 6 µl of RT buffer, 2 µl of 0.1 M DTT (Invitrogen), 0.1 µl of ribonuclease inhibitor (Invitrogen) and 1 µl of M-MLV reverse transcriptase (Invitrogen). The reaction was conducted in 37 °C for 1 h.

#### Nested PCR

For nested PCR (nRT-PCR) were used two pairs of primers (1: 5′-CTCTTTCGACACTCGTCGAGG-3′, 2: 5′– GCGTTTGCT(C,T)CGGA-3′ and 3: 5′-CCTTTCAG(A,G)ATGGCCTT-3′, 4: 5′-CGGA(C,T)AGCATTAGCAGCG-3′) for the 5′-NCR and the 5′-terminus of the C protein coding region, which are highly conserved among the TBEV isolates (Ramelow et al. 1993). PCR reactions were conducted in a reaction mixture containing: 2.0 µl of cDNA template, 0.5 U (0.5 µl) of *Taq* polymerase RUN (A&A Biotechnology), 2.0 µl of 10 × PCR reaction buffer with Mg^++^, 2.0 µl of 2.5 mM dNTPs mixture (MBI Fermentas), 0.5 µl of 10 µM primer (1, 2 in the first round and 3 and 4 in the second) and nuclease free water. In this assay, the size of the first round amplification product was 175 nucleotides and that of the second round amplification was 128 nucleotides. For the second round, 1 µl of amplicon of the first round was used. All PCRs were conducted in 20 µl volume and under the same conditions: 15 min at 95 °C for initial denaturation, followed by 38 cycles: 1 min at 92 °C denaturation, 1 min at 37 °C annealing, 2 min at 72 °C extension and 7 min final extension at 72 °C (Sparagano et al. 1999).

Amplification products were analysed by electrophoresis in 1.5 % agarose gel stained with ethidium bromide. One positive (low pathogenic TBEV Langat strain) and two negative (sterile RNase-free water instead of tick RNA after the RT reaction and DDW instead of cDNA in nested PCR reactions) controls were run with each PCR reaction. To prevent contamination, work surfaces and apparatus were treated with RNase Zap (Ambion, Austin, TX, USA) which completely removes contamination with RNase. We used also one-use disposable tips with filters (PCR-clean/dualfilter/sterile) (Eppendorf, Germany) and disposable one-use test tubes (PCR-clean, free of detectable human DNA, DNase, RNase, PCR inhibitor) (Starlab, Hamburg, Germany).

#### DNA sequencing

Amplicons (128 bp) of randomly selected positive samples were removed from the gel under UV exposure and purified with the Gel Out purification kit (A&A Biotechnology). DNA sequencing reactions were performed with the ABI PRISM 310 Genetic Analyzer (Applied Biosystems, USA) with standard procedure described by the manufacturer. Electropherograms were manually inspected and corrected using ProSoftware (GeneStudio, Suwanee, GA, USA). Sequences were edited and compared with representative gene sequences deposited in GenBank database using NCBI BLAST software (http://www.ncbi.nlm.nih.gov/BLAST) (U.S. National Institutes of Health, Bethesda, MD, USA).

#### Statistics

Descriptive statistics were performed by Pearsons Chi square test using Statistica 10 software.

## Results

Altogether 471 adult *D. reticulatus* ticks (316 females and 155 males) were collected and tested for the presence of TBEV RNA. Of them 10 were positive in the nRT-PCR assay (Table 1). Positive results were noted in all investigated sites. The overall infection rate of ticks with TBEV in all collection sites was calculated as 2.12 %, ranging from 0.99 % (Kosewo Górne) to 12.5 % (Trzcianne). The percent of infected females and males was similar and was equal to 2.17 and 1.98 %, respectively (Table 1). Higher percentage of infected ticks was noted in the urban area (Warsaw) compared to natural areas, not transformed by human activity (national and landscape parks) (3.12 vs. 1.96 %, respectively). However, this difference was not statistically significant.

**Table 1:** Prevalence of Tick-borne encephalitis virus RNA in *Dermacentor reticulatus* ticks collected in North-Eastern Poland and the city of Warsaw (CS Poland)

*D. reticulatus* No. infected/no. examined (% infected)			
Collection site (district)/Geographical coordinates	Female	Male	Total
*Mazowieckie voivodeship*			
Warsaw (Warsaw) 52^o^13′56″N, 21^o^00′30″E	1/46 (2.17)	1/18 (5.55)	2/64 (3.12)
*Podlaskie voivodeship*			
Białowieża (Białowieża) 52^o^42′04″N, 23^o^52′10″E	2/102 (1.96)	0/24 (0.0)	2/126 (1.58)
Gugny (Trzcianne) 53^o^21′00″N, 22^o^35′29″E	3/63 (4.76)	0/9 (0.0)	3/72 (4.16)
Trzcianne (Trzcianne) 53^o^20′00″N, 22^o^41′00″E	1/5 (20.0)	0/3 (0.0)	1/8 (12.5)
*Warmińsko-Mazurskie voivodeship*			
Kosewo Górne (Mrągowo) 53^o^48′14″N, 21^o^23′20″E	0/100 (0.0)	2/101 (1.98)	2/201 (0.99)
Total	7/316 (2.21)	3/155 (1.93)	10/471 (2.12)

**Fig. 2:**
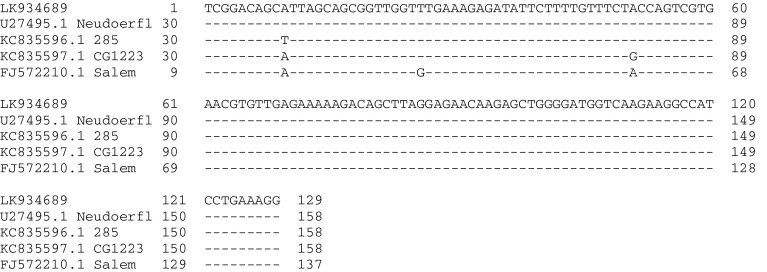
Comparison of the obtained sequence (LK934689) of Tick-borne encephalitis virus from *Dermacentor* *reticulatus* with the sequences deposited in GenBank

Sequencing of PCR products was performed on 4/10 randomly selected positive samples. All of the obtained sequences (Dr172-222) (ENA European Nucleotide Archive acc. no. LK934689) had 100 % similarity to each other and to the following Tick-borne encephalitis virus sequences deposited in GenBank: strain Ljubljana I (Acc. No. IQ654701.1), Kumlinge A52 (Acc. No. GU183380.1) and Neudoerfl (Acc. No. U27495.1|TEU27495). They had 99 % similarity to the sequence TBEV strain 285 (Acc. No. KC835596.1) (transitions A–T in position 40), CG1223 (Acc. No. KC835597.1) (transitions A–G in position 80) and Salem (Acc. No. FJ572210.1) (transition T–G in position 34) (Fig 2).

## Discussion

In this study, the infection rate of *D.* *reticulatus* ticks with TBEV across the whole investigated area was 2.12 %. It is a significantly lower score than the one derived from the same species of ticks from Lublin region (SE Poland) where it was equal to 10.8 % and, in contrast to this study, it was slightly lower in males than in females (11.8 and 10.3 %, respectively) (Wójcik-Fatla et al. 2011). On the other hand, studies on *D. reticulatus* from Podlaskie voivodeship, an endemic TBE area, conducted using real-time PCR yielded the MIR (minimum infection rate) score of only 0.33 % (Kondrusik et al. 2010). It has to be emphasized, however, that according to Stefanoff et al. (2013) the results of assays for the presence of TBEV in ticks tend to be higher when nested RT-PCR instead of real-time PCR is used as the method for viral RNA detection. Furthermore, in the current study, the level of TBEV infection was higher in *D. reticulatus* than so far noted in *I. ricinus*. The infection level of the latter species from Lublin region determined by virus isolation was 1.8 % (Cisak et al. 2002) while determined by nested RT-PCR was 1.6 % (Wójcik-Fatla et al. 2011). Research in north-western Poland, conducted by nested RT-PCR, showed that 7.9 % out of 177 tested *I. ricinus* tick pools were positive for TBEV presence, although the MIR was not calculated (Makówka et al. 2009). According to the presented data, however, one could estimate MIR at 0.58 %. Comparing the results of this study, conducted in Warmińsko-Mazurskie and Podlaskie voivodeships, to a similar study relating to *I. ricinus,* conducted in the same areas and with the same methods (Biernat et al. 2014 accepted for publication), the average percentage of infected ticks was slightly higher in *D. reticulatus* (1.96 %) than in *I. ricinus* (1.39 %). In our study, a special attention is drawn to the 3.12 % prevalence of infected ticks from urban area (Warsaw)—which is statistically comparable to the estimated prevalence of 1.96 % in natural areas (Podlaskie and Warmińsko-Mazurskie voivodeships). High abundance of this tick species in urban area was noted also in the urban area of Lublin and Lubartów (Podlaskie voivodeship), directly adjacent to housing estates. *D. reticulatus* population there was comparable in number with the habitats described thus far as the most optimal for this species (Biaduń et al. 2007; Biaduń 2011). Ticks have always been a part of urban fauna, especially in suburban areas. The urbanization and human activities connected with it may often positively influence the occurrence and abundance of ticks (Upensky 2014). It can be assumed that in this case, hosts of adult *D. reticulatus* are probably dogs which may also carry infected ticks from endemic to non-endemic areas and into close vicinity of humans. Tick-borne diseases like TBE in dogs have increased in incidence and clinical importance in recent years (Leschnik et al. 2002). Due to the host range, the possibility of human infection with TBEV by *D. reticulatus* bite is low. These ticks may, however, support the circulation of the virus in the environment due to transstadial and transovarial transmissions (Alekseev et al. 1996; Naumov et al. 1980) which allow for the maintenance of the virus in ticks for many generations, even in the absence of infection-susceptible mammals in the environment.

No differences were found between the isolates from *D. reticulatus* and *I. ricinus* from Poland. Sequences of partial C protein coding region (128 bp) recovered from *D. reticulatus* in this study showed 100 % homology to known strains of Western Tick-borne Encephalitis Virus (TBEV-W) and they were 100 % identical with the corresponding sequences of TBEV obtained so far from both *D. reticulatus* and *I. ricinus* collected in the Lublin region (Eastern Poland) (Wójcik-Fatla et al. 2011) and *I. ricinus* from North-Eastern Poland (Biernat et al. 2014 accepted for publication).

The ability of ticks to transmit numerous human and animal pathogens, including TBEV, and the presence of many reservoir hosts create persistent danger for human populations and domestic animals also in urban and suburban areas. This results in the need of protection of humans and domestic animals from ticks bites.
